# Challenges in establishing telehealth care during the COVID-19 pandemic in a neglected HTLV-1-infected population in northeastern Brazil

**DOI:** 10.1371/journal.pntd.0008922

**Published:** 2020-12-31

**Authors:** Bernardo Galvão-Castro, Maria Fernanda Rios Grassi, Aidê Nunes, Ana Karina Galvão-Barroso, Ana Verena Galvão-Castro, Monique Lírio, Adriele Ribeiro, Thiago de Faria Junqueira, André Luís Silva, Maíara Cerqueira, Sonia Lucia Rangel, Thessika Hialla Almeida Araujo, Ney Boa-Sorte, Inês Dourado, Humberto Castro-Lima, Maria Luísa Carvalho Soliani

**Affiliations:** 1 Escola Bahiana de Medicina e Saúde Pública, Salvador, Brazil; 2 Laboratório Avançado de Saúde Pública, Instituto Gonçalo Moniz, Fundação Oswaldo Cruz, Salvador, Brazil; 3 Instituto de Saúde Coletiva, Universidade Federal da Bahia, Salvador, Brazil; Federal University of Ceará, Fortaleza, Brazil, BRAZIL

## Case presentation

### The impact of the COVID-19 pandemic in Bahia and Brazil

The novel Severe Acute Respiratory Syndrome Coronavirus 2 (SARS-CoV-2) and its corresponding Coronavirus Disease 2019 (COVID-19) first appeared in China in December 2019 as a cluster of atypical severe pneumonia cases [1]. Rapid viral spread occurred worldwide, causing tremendous impacts on human health and global economies. The World Health Organization (WHO) declared COVID-19 as a public health emergency of international importance on January 30, 2020, followed by pandemic status on March 11 [2]. Although SARS-CoV-2 reached Latin America later than Europe, the number of cases has quickly and steadily risen in the region. The first case of SARS-CoV-2 infection in Brazil and in Latin America was reported in São Paulo on February 25, 2020. The virus has since spread throughout the country, first in the Southeast (São Paulo and Rio de Janeiro) and then the North (Amazonas and Pará) and the Northeast (Pernambuco and Ceará). In the initial phase of the epidemic, Brazil presented 1 of the highest rates of SARS-CoV-2 transmission (*R*0 = 2.81) in the world [3]. Bahia, the most populous state in the northeast, with the fourth largest population size in Brazil, reported its first case of SARS-CoV-2 infection on March 6.

As of July 15, 2020, SARS-CoV-2 is present in 188 countries, with 13,520,403 cases of infection, resulting in 582,784 deaths [4]. At the time of this report, Brazil currently ranks second in the number of cases and deaths due to COVID-19 worldwide, with an accumulated 1,933,655 confirmed cases and 74,336 deaths [4]. In an attempt to contain the SARS-CoV-2 epidemic, the Brazilian Health Ministry recognized the epidemic as a public health emergency of national importance on February 2, and the Brazilian senate approved legislation (law no. 13,97916, *Lei da Quarentena*) on March 6, allowing states and municipalities to implement discretionary social isolation and distancing measures [5]. However, Brazil has endured an uncoordinated national government response [6]; the country’s structural complexities, such as social and regional inequities, in addition to quarantine measures being only partially implemented, have caused the epidemic to rapidly advance, especially among disadvantaged and marginalized populations [7]. The decentralized efforts implemented by state and municipal governments, ranging from voluntary social isolation to partial lockdowns, has allowed for a flexible approach in accordance with regional conditions, in coordination with public healthcare services provided by the country’s National Health System (Sistema Único de Saúde [SUS]) [8].

### Current status of HTLV infection in Bahia (northeastern Brazil)

**Fig 1:**
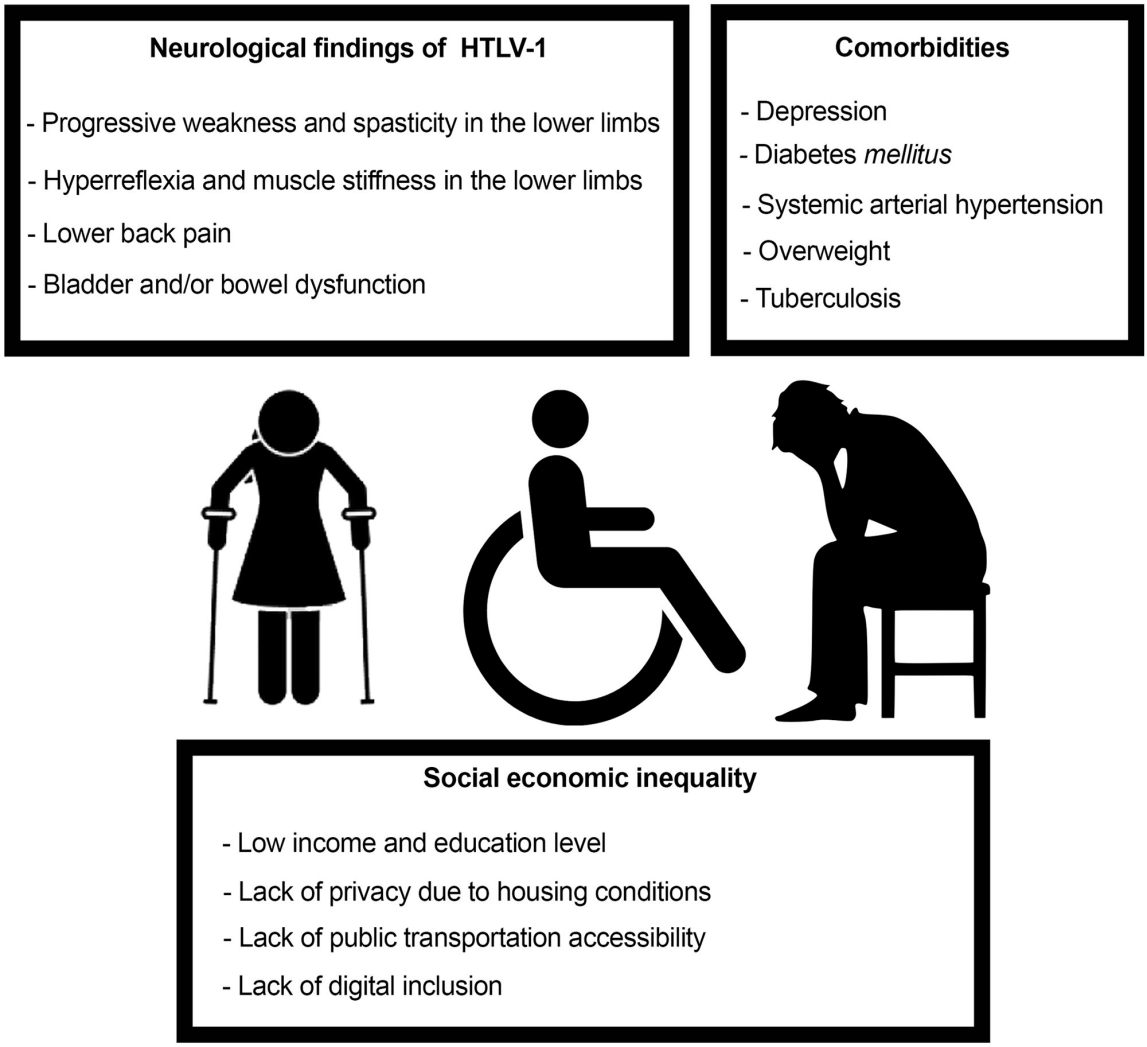
Clinical signs and symptoms, associated comorbidities, and socioeconomic profile of people living with HTLV-1 in Brazil. HTLV-1, human T-cell leukemia virus type-1. *Images obtained from* http://www.publicdomainpictures.net/ and https://openclipart.org/.

Human T-cell leukemia virus type-1 (HTLV-1), also known as human T-lymphotropic virus type 1, was the first retrovirus to be isolated in humans and is etiologically linked with adult T cell leukemia/lymphoma (ATLL), tropical spastic paraparesis/HTLV-1-associated myelopathy (TSP/HAM), uveitis, and infective dermatitis. In addition, several other diseases have been associated with HTLV-1 infection, such as polymyositis, sinusitis, bronchoalveolar pneumonia, keratoconjunctivitis sicca, bronchiectasis, and urinary disorders [9]. Psychiatric and/or psychological disorders, such as depression and anxiety, have also been reported affected patients, heavily impacting the quality of life of people living with HTLV-1 [10] (Fig 1).

It is estimated that at least 5 to 10 million people harbor the virus worldwide. With around 800,000 infected individuals, Brazil represents the largest number of HTLV-1 carriers on the American continent [9]. The state of Bahia is 1 of the most affected by HTLV-1 in the country, with approximately 130,000 infected persons [11]. In Salvador, the state capital, a population-based study found that 1.76% of the city’s population was infected with HTLV-1. The highest prevalence was found in females, with approximately 10% of women aged 50 years or older being infected [12]. HTLV-1 infection occurs more frequently among individuals with low socioeconomic status and low education levels. Diseases associated with the virus, such as HAM/TSP and ATLL, rank among the most neglected in Brazil [13,14].

### Multidisciplinary care for HTLV-1 patients and telehealth in Brazil

**Fig 2:**
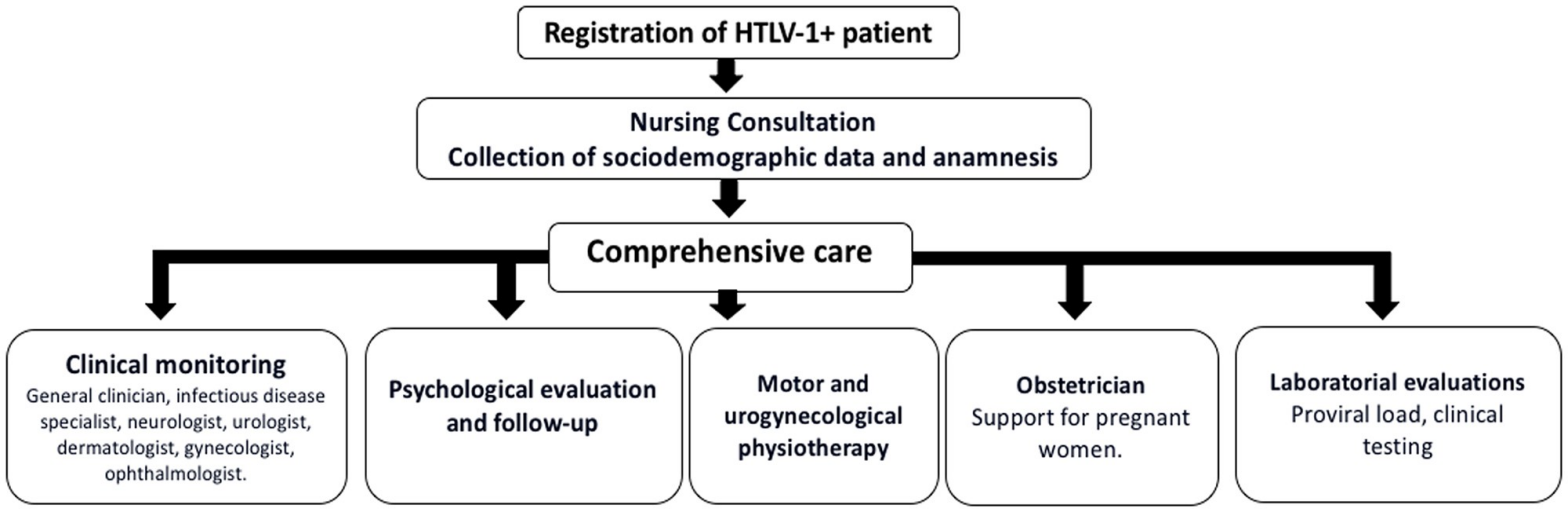
Biopsychosocial comprehensive care for HTLV-1-infected individuals followed at the CHTLV in Salvador, Brazil. CHTLV, Integrated Multidisciplinary HTLV Center; HTLV-1, human T-cell leukemia virus type-1.

In an effort to provide integrated and multidisciplinary care to HTLV-1-infected patients, the Integrated Multidisciplinary HTLV Center (CHTLV) was established in Salvador in 2002 at the Bahiana School of Medicine and Public Health (Escola Bahiana de Medicina e Saúde Pública—EBMSP). This outpatient clinic provides comprehensive biopsychosocial care to the public, with support provided for patients by SUS (Fig 2). A total of 2,160 HTLV-1 patients have been registered at CHTLV since 2002, with approximately 50% being regularly seen at least once a year. Most infected patients are poor women who face great difficulty in terms of arriving for consultations at CHTLV, due to limiting physical conditions and a lack of public transportation accessibility.

In recognition of the importance of telehealth in improving access to healthcare, EBMSP began a program to train healthcare staff in health informatics and telehealth services in 2017. In Brazil, prior to the COVID-19 pandemic, the implementation of telehealth faced resistance from healthcare professionals, as well as national and state medical boards and healthcare councils. Moreover, complex legal and technological requirements hindered the widespread adoption of telehealth services.

### Why implement telehealth for patients infected with HTLV-1 during the COVID-19 epidemic?

With the advent of the COVID-19 pandemic and governmental guidelines recommending social distancing, EBMSP implemented a contingency plan for employees, students, and patients [15], which made it impossible for patients to be seen at CHTLV. Despite the fact that no reports have indicated that COVID-19 may be more severe in patients infected with HTLV-1, special care recommendations have been published for this group, since this infection predominantly affects older individuals for whom comorbidities such as diabetes, systemic arterial hypertension, and overweight are frequent [16]. Moreover, it has been reported that HTLV-1-infected individuals may present some degree of immunosuppression, since several infectious diseases, such as tuberculosis, strongyloidiasis, and scabies, are more frequent or more severe in these individuals [9].

### How to implement telehealth consultations for HTLV-1-infected patients during the COVID-19 epidemic?

The COVID-19 pandemic forced changes in the Brazilian healthcare landscape, including the simplification of legislation regarding telehealth, allowing healthcare providers to use online platforms to provide assistance during this period. Patients who were being regularly followed at CHTLV and who had previously scheduled appointments were contacted by telephone to ascertain their interest in telehealth consultations. These consultations then began to be scheduled with members of a multidisciplinary team composed of nurses, infectious disease specialists, neurologists, physicians, physiotherapists, and psychologists, with the aim of providing integrated care whenever possible. While consultations are scheduled to occur weekly, patients can also schedule additional consultations if necessary. Team members contact patients by video or audio conferencing. Each patient’s identity is confirmed at the beginning of every consultation by stating his/her full name, date of birth, and mother’s full name. Access to patient electronic health records is performed via an exclusive password to ensure patient confidentiality. All patients provide verbal consent and are informed of the temporary nature of this mode of consultation. During the appointment, the patient’s recent medical history is reviewed by a physician and nurse, and health guidelines and planning are communicated based on each patient’s needs. To obtain medical-related assistance from governmental agencies, prescriptions and reports are either provided digitally or printed copies are delivered afterwards to patients or their family members. During consultations with a psychologist, specific questions address social distancing, as well as type of routine, family/relative/friend support network, quality of sleep, and mood. In consultations with a physiotherapist, functional capacity is assessed by the application of the Osame and Kurtze scales [17] when possible, and changes in urinary function are evaluated using a bladder diary. Patients are also advised to perform physical activity.

### How were HTLV-1 patients monitored via telehealth during the COVID-19 pandemic?

The presence of respiratory symptoms was investigated in all patients, who received instructions on preventative measures against SARS-CoV-2 infection. In addition, the importance of continuing treatment for comorbidities was reinforced. Patients were also instructed to receive vaccinations against seasonal influenza. All followed clinical cases were discussed in a virtual meeting involving all multidisciplinary team members at the end of each week. Each patient’s therapeutic approach was coordinated by the team.

From April 14 to July 7, 2020, 132 patients were scheduled for appointments; 75 could not be reached by phone. Five patients preferred not to have telehealth consultations. In all, 52 patients (39.4%) patients received care remotely, with a total of 138 consultations performed. Most patients (*n* = 29, 55.7%) were seen by at least 2 specialists on the day in which their appointment was scheduled, 8 patients (15.4%) were seen by 3 specialists, 12 (23.1%) by 4, and 3 (5.8%) patients were seen simultaneously by 5 team members. Patients were seen by a nurse 27 (19.6%) times, an infectious disease specialist 25 (18.1%) times, a neurologist 28 (20.3%) times, a physiotherapist 32 (23.2%) times, and a psychologist 26 (18.8%) times.

### What are the socioeconomic and clinical profiles of the HTLV-1 patients being monitored via telehealth during the COVID-19 pandemic?

Almost all patients (98%) lived in the city of Salvador (Bahia, Brazil), and 46% were diagnosed with HAM/TSP. The age of the patients ranged from 29 to 72 years (mean: 53 [SD 13.4]), 73% were female, 84.6% self-reported skin color as black or mixed race, 73% studied for 8 years or more, and half earned the equivalent of a single monthly Brazilian minimum wage (US$200). In addition, 2 patients informed team members that they had tested positive for COVID-19 by molecular diagnosis; these individuals are being followed closely to evaluate any associated impacts.

## Case discussion

Despite the difficulties imposed by the COVID-19 pandemic, it was possible to implement telehealth consultations as a way to follow patients regularly seen at CHTLV. The remote care provided by the multidisciplinary team allowed for the early diagnosis of complications and stimulated adherence to treatment in HTLV-1-infected patients. In addition, telehealth consultations could help alleviate the psychological distress caused by social distancing. At the end of each scheduled appointment, all patients were asked to provide feedback regarding the novel format; all reported satisfaction with the novel consultation format. Our efforts resulted in strengthening the bonds between patients and the multidisciplinary team, especially considering the impact of social isolation measures. To more comprehensively evaluate the effectiveness of the telehealth program, an objective questionnaire should be applied.

Despite the organizational efforts of the multidisciplinary team in establishing telehealth consultations, the number of patients seen was fewer than expected. The main obstacles identified in carrying out teleconsultations in a lower income population were (i) inadequate access to computers and smartphones with internet access that allowed for a stable interaction by video call; (ii) frequent changes in telephone contact numbers; (iii) difficulties in using technology; and (iv) lack of privacy during consultations due to housing conditions (i.e., high household density).

It is important to recognize that telehealth consultations represent an important tool to provide complementary care for patients; however, some physical examinations may not be possible to perform remotely, especially first-time patient visits. It is important that clinicians discuss these limitations with patients and inform suitable alternatives. Looking to the future, telehealth consultations may continue to be useful in monitoring patients who live in remote areas or who have physical and/or financial limitations, which restricts their ability to travel for in-person appointments. Based on our initially positive results, we plan to continue to offer telehealth services to HTLV-1-infected individuals registered at CHTLV in the post-pandemic period under the framework provided by SUS and EBMSP. However, it will be important for other centers interested in implementing telehealth programs to secure financial support to ensure the continuity of their services.

**Key Learning Points**

- It is possible to implement a telehealth program to serve a vulnerable human T-cell leukemia virus type-1 (HTLV-1)-infected population.
- Patients must have at least minimal access to the internet and a smartphone, computer, or tablet available.
- Privacy at the time of the consultation is important to maintaining trust between caregivers and patients.
- Telehealth can support the monitoring of existing comorbidities and aid in the surveillance of respiratory symptoms, allowing for the early detection of Coronavirus Disease 2019 (COVID-19).
- Telehealth services were important in the context of social distancing implemented during the health crisis.
